# Transthyretin amyloid cardiomyopathy in patients with unexplained increased left ventricular wall thickness

**DOI:** 10.1007/s10554-024-03158-z

**Published:** 2024-06-10

**Authors:** Katarzyna Holcman, Magdalena Kostkiewicz, Wojciech Szot, Bogdan Ćmiel, Krystian Mróz, Agnieszka Stępień, Katarzyna Graczyk, Ewa Dziewięcka, Aleksandra Karabinowska-Małocha, Zuzanna Sachajko, Piotr Podolec, Paweł Rubiś

**Affiliations:** 1 Department of Cardiac and Vascular Diseases, Jagiellonian University Medical College, John Paul II Hospital, Pradnicka 80, 31-202 Krakow, Poland; 2 Department of Nuclear Medicine, John Paul II Hospital, Krakow, Poland; 3 Department of Hygiene and Dietetics, Jagiellonian University Medical College, Krakow, Poland; 4 Faculty of Applied Mathematics, AGH University of Science and Technology, Krakow, Poland; 5 Students Scientific Group of Heart Failure, Department of Cardiac and Vascular Diseases, Jagiellonian University Medical College, Krakow, Poland

**Keywords:** Transthyretin amyloidosis, ATTR, Hypertrophic cardiomyopathy, DPD, SPECT, Amyloid cardiomyopathy

## Abstract

Amyloid cardiomyopathy (CA) was previously considered a rare disease; however, rapid advancements in imaging modalities have led to an increased frequency of its diagnosis. The aim of this prospective study was to assess the prevalence and clinical phenotype of transthyretin amyloidosis (ATTR) cardiomyopathy in patients exhibiting unexplained increased left ventricular (LV) wall thickness. From 2020 to 2022, we enrolled 100 consecutive adults with unexplained increased LV wall thickness in the study. The analysis included clinical data, electrocardiography, transthoracic echocardiography, single-photon emission computed tomography/computed tomography with 3,3-disphono-1,2-propanodicarboxylic acid, genetic testing. Overall, 18% of patients were diagnosed with CA, comprising 5% with light-chain amyloidosis, and 12% with ATTR. To evaluate associations with the ATTR diagnosis, a LOGIT model and multivariate analysis were applied. Notably, age, polyneuropathy, gastropathy, carpal tunnel syndrome, lumbar spine stenosis, low voltage, ventricular arrhythmia, LV mass, LV ejection fraction, global longitudinal strain (GLS), E/A, E/E′, right ventricle (RV) thickness, right atrium area, RV VTI, TAPSE, apical sparing, ground glass appearance of myocardium, thickening of interatrial septum, thickening of valves, and the “5–5–5” sign were found to be significantly associated with ATTR (p < 0.05). The best predictive model for ATTR diagnoses exhibited an area under the curve of 0.99, including LV mass, GLS and RV thickness. This study, conducted at a cardiology referral center, revealed that a very considerable proportion of patients with unexplained increased LV wall thickness may suffer from underlying CA. Moreover, the presence of ATTR should be considered in patients with increased LV mass accompanied by reduced GLS and RV thickening.

## Introduction

Unexplained left ventricle (LV) thickening may be observed in the course of various conditions such as sarcomeric hypertrophic cardiomyopathy (HCM) or metabolic, neuromuscular, endocrine, and infiltrative disorders [1, 2]]. Transthyretin amyloidosis (ATTR) presents a complex clinical picture, making it a challenging disease to address, both in terms of initial evaluation and subsequent treatment. It develops secondary to the deposition of misfolded, insoluble transthyretin (TTR) fibrils in the extracellular matrix of various organs and tissues, including the heart. There are two distinct forms of ATTR: acquired wild-type ATTR (ATTRwt), and hereditary ATTR (ATTRm), inherited in an autosomal dominant pattern with variable penetrance [3]. While previously considered rare outside of endemic areas, it is currently diagnosed more frequently due to an increase in medical awareness and significant developments in non-invasive diagnostics [4]. Scintigraphy using bone-avid tracers such as 3,3-disphono-1,2-propanodicarboxylic acid (DPD), methylenediphosphonicacid (MDP), and pyrophosphate (PYP), has become a fundamental technique for identifying ATTR patients [5]. Recent years have yielded more data justifying the application of imaging-based diagnostic approaches, and as a result, their use was included in the Working Group on Myocardial and Pericardial Diseases of the European Society of Cardiology (ESC) position statement [6]. Importantly, recent data has shown a higher prevalence of ATTR in specific patient subpopulations, with rates reaching 13% in heart failure with preserved left ventricular ejection fraction (HFpEF), 5% in patients with HCM, and 16% in severe aortic stenosis [7–11]. As this progressive disease carries a poor survival prognosis, ongoing efforts are focused on developing emerging ATTR therapies [12–16]. Hence, accurate diagnosis may be vital in improving outcomes for this group of patients. This prospective, single-center study sought to assess the prevalence and clinical phenotype of ATTR cardiomyopathy in patients with unexplained increased LV wall thickness.

## Methods

### Study population and study protocol

This prospective study was conducted in a tertiary cardiac center from 2020 to 2022. In total, 117 potentially eligible participants were screened, and 100 consecutive adults were enrolled, having met inclusion criteria. Exclusion criteria for the study encompassed patients with drug-resistant hypertension, resulting in eight individuals being omitted, the presence of severe aortic stenosis and severe aortic regurgitation led to the exclusion of five and two patients, respectively, and pre-existing infiltrative disorders, which accounted for the exclusion of a further two participants. The analysis encompassed clinical data, biochemical analysis, free light chain blood immunoglobulins and urine immunofixation, electrocardiogram (ECG), 24-h Holter monitoring, transthoracic echocardiography (TTE), 6-min walking test, and single-photon emission computed tomography/computed tomography (SPECT/CT) with Technetium 99 m and DPD tracer ([99mTc]Tc-DPD). In *selected* cases, where the noninvasive algorithm provided unequivocal results, a cardiac or soft tissue biopsy was performed to confirm the final diagnosis [4]. Patients with grade 1–3 [99mTc]Tc-DPD cardiac uptake in scintigraphy were subjected to genetic testing by an amplicon-based next-generation TTR sequencing approach. Patients with positive free light chain blood immunoglobulins or urine immunofixation were referred to a hematology specialist and underwent bone marrow biopsy. The inclusion criteria required participants to be over 18 years of age, provide written informed consent, and have LV wall thickness greater than 15 mm (mm) as assessed by TTE. Exclusion criteria included pregnancy, lactation, preexisting causes of LV hypertrophy (LVH) such as drug-resistant hypertension, severe aortic stenosis with aortic valve area (AVA) < 1.0 cm^2^, severe aortic regurgitation, severe mitral stenosis, or other previously diagnosed preexisting infiltrative disorders. The study population was divided into two groups: those diagnosed with ATTR (group 1, n = 12), and those without ATTR (group 2, n = 88).

### Echocardiography

Echocardiograms were conducted using a Philips EPIQ7 device (the Netherlands) by experienced operators who were blinded to the patients’ final diagnoses, in accordance with current guidelines [5, 17]. The evaluation included planar measurements, M-mode, continuous Doppler, pulsed Doppler, tissue Doppler, and color Doppler in standard views. Longitudinal LV strain curves were obtained manually in the apical 2-, 3-, and 4-chamber projections. The global LV longitudinal strain (GLS) value (calculated using the peak negative instantaneous average of the 18 longitudinal segmental strains) was also assessed. Additionally, the pattern displayed on the longitudinal strain bullseye map for individual segments was examined [18]. Ground glass appearance was defined as the visual echocardiographic characteristic of the myocardium with a diffuse, increased echogenicity [5]. Valvular and interatrial septum thickening was defined as a value beyond 5 mm [5]. The apical sparing pattern was defined by a ratio of the average longitudinal strain of the apical segments to that of the basal and mid segments above a 1.0 threshold [5, 18]*.*

### Scintigraphy

The image acquisition procedures adhered to the current recommendations [5]. In summary, Technetium 99 m (370–740 MBq) and DPD tracer (TECEOS, CIS BIO) were administered for the procedure. The protocol included whole-body scans 2–3 h following intravenous radiotracer administration. The planar scintigraphic scans were classified based on the Perugini semi-quantitative scale, which uses the following grades: grade 0 (no myocardial uptake and normal bone uptake), grade 1 (myocardial uptake less than rib uptake), grade 2 (myocardial uptake equal to rib uptake), and grade 3 (myocardial uptake greater than rib uptake with mild/absent rib uptake) [5, 19]. Computed tomography (CT) attenuation-corrected and non-corrected single photon emission tomography (SPECT) images were evaluated in the coronal, transaxial, and sagittal planes, as well as in tridimensional maximal-intensity projection cine mode. The planar and hybrid SPECT/CT images were independently assessed by two experienced nuclear medicine specialists, blinded to clinical information.

### Statistical analysis

Conformity with a normal distribution was assessed with the Shapiro–Wilk test. For continuous variables, a comparison between groups was conducted with Student’s *t*-test for mean values. Variables without a normal distribution were analyzed using the Mann–Whitney *U* test. Categorical variables were analysed using the χ^2^ test. The Fisher’s exact test was applied to categorical data in all instances where the number of observations in any subgroup was fewer than five. A generalized linear model (LOGIT model) was applied to calculate odds ratios (OR) and 95% confidence intervals (CI) for the endpoint, defined as ATTR diagnosis. A multivariate analysis was performed to investigate the associations of selected variables with the endpoint, including information on the clinical data, laboratory results, electrocardiographic and echocardiographic parameters. P-values below 0.05 were deemed to be statistically significant. The analysis involved identifying a model that predicts a diagnosis of ATTR in this population, including the assessment of the area under the curve (AUC) value and the receiver operating characteristic (ROC) curve. Statistical analyses were performed using Statistica 13.0 and MedCalc software. The data underlying this article will be shared upon reasonable request to the corresponding author.

### Compliance with ethical standards

Written informed consent was obtained from all of the participants enrolled in the study. All the procedures performed were in accordance with the ethical standards of the local Ethics Committee and with the 1964 Helsinki declaration, and its later amendments, or comparable ethical standards.

## Results

**Fig. 1:**
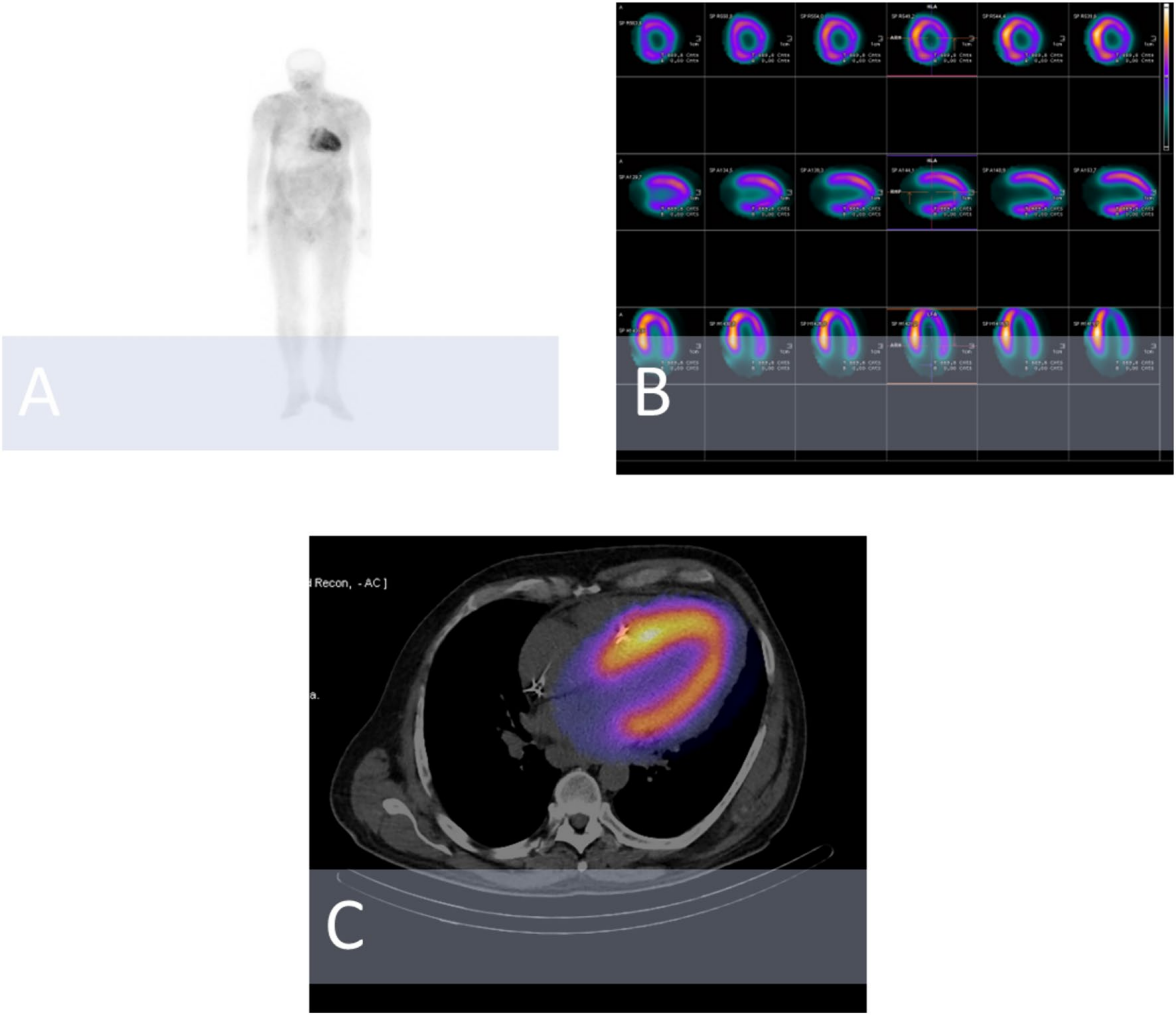
Findings in the course of hereditary cardiac transthyretin amyloidosis. **A** planar whole-body scintigraphy with [99mTc]Tc-DPD (grade 3). **B** SPECT imaging with [99mTc]Tc-DPD (after attenuation correction). **C** hybrid SPECT/CT imaging. *CT* computed tomography, *DPD* 3,3-disphono-1,2-propanodicarboxylic acid, *SPECT* single-photon emission computed tomography

Between 2020 and 2022, a total of 100 consecutive patients (mean age 59.9 ± 13.9 years) were enrolled in the study. The characteristics of the study population are presented in Table 1. In the entire population, there was a predominance of male participants. Overall, among the patients 32% were diagnosed with atrial fibrillation (AF). The diagnoses of polyneuropathy, lumbar spine stenosis and carpal tunnel syndrome were observed in 6%, 5% and 3% of patients, respectively. The distribution of echocardiographic and nuclear findings within the groups are presented in Tables 2 and 3. Echocardiography revealed a mean LV maximum wall thickness of 18.8 ± 4.4 mm and an LV mass value index value of 179 ± 46 g/m^2^. Taken as a whole, the mean LV ejection fraction (LVEF) value reached 55 ± 16%, and the GLS value was − 15.7 ± − 5.7%. Overall, SPECT/CT imaging detected cardiac tracer uptake in 13% of patients (Fig. 1).

**Table 1:** Baseline demographic, clinical and laboratory characteristics of the study population divided into two groups: those diagnosed with transthyretin amyloidosis (group 1), and those without transthyretin amyloidosis (group 2)

Variable	Group 1 * (n = 12)	Group 2 * (n = 88)	p Value
Male gender	9 (75%)	58 (66%)	0.74
Body mass index (kg/m^2^)	25.5 ± 3.7	27.5 ± 5.7	0.06
Age (years)	69 ± 12	60 ± 13	**0.007**
Diabetes mellitus	3 (25%)	21 (24%)	1.00
Cardiovascular implantable electronic device	6 (50%)	10 (11%)	** < 0.001**
Amyloidosis type			
ATTRm ATTRwt AL Other types	4 (33%) 8 (67%) 0% 0%	0% 0% 5 (6%) 1 (1%)	
Transthyretin variants present	Phe53Leu (25%) Ala101Val (8%)	0%	** < 0.001**
Polyneuropathy	4 (33%)	2 (2%)	**0.005**
Gastropathy	3 (25%)	0%	**0.001**
Carpal tunnel syndrome	3 (25%)	0%	**0.001**
Chronic kidney disease	6 (50%)	26 (29%)	0.17
Bicep tendon rupture	1 (8%)	1 (1%)	0.27
Lumbar spine stenosis	4 (33%)	1 (1%)	** < 0.001**
Atrial fibrillation	6 (50%)	26 (29%)	0.17
Weight loss over the previous 6 months	2 (16%)	1 (1%)	**0.04**
Positive family history for amyloidosis	2 (16%)	0%	**0.01**
NYHA class III-IV	7 (58%)	32 (36%)	**0.04**
Systolic blood pressure (mmHg)	110 ± 15	127 ± 23	**0.01**
Heart rate (beats per minute)	66 ± 12	67 ± 13	0.91
Peripheral edema	4 (33%)	19 (21%)	0.46
Hematocrit (%)	38 ± 5	41 ± 5	0.68
Creatinine (mg/dl)	96 ± 42	90 ± 71	0.99
Aspartate transaminase (U/l)	22 ± 8	25 ± 12	0.38
NT-proBNP (pg/ml)	5071 (1805, 9600)	1108 (222, 2500)	**0.002**
Cardiac troponin T (ng/ml)	0.05 (0.04, 0.11)	0.02 (0.01, 0.05)	**0.004**
Albumin (g/l)	35 ± 3	38 ± 4	0.15
Bence-Jonce protein present	0%	6 (7%)	1.00
MGUS	1 (8%)	4 (4%)	0.48
ECG—low voltage	8 (67%)	7 (8%)	** < 0.001**
ECG—pseudo-infarct pattern	6 (50%)	50 (57%)	0.26
Atrioventricular block	6 (50%)	11 (12%)	**0.002**
Ventricular arrhythmia (more than 1000 premature heart beats in 24 h, bigeminy, trigreminy, accelerated idioventricular rhythm, ventricular tachycardia)	10 (83%)	17 (19%)	** < 0.001**
6-min walking test distance (m)	270 ± 161	390 ± 132	0.18

*The data is given as a number (percentage) for categorical data, and as a mean value ± one standard deviation or median (IQR) for continuous variables

*AL* light chain amyloidosis, *ATTRwt* transthyretin amyloidosis wild-type, *ATTRm* hereditary transthyretin amyloidosis, *ECG* electrocardiogram, *NYHA* New York Heart Association class, *NT-proBNP* N-terminal pro-brain natriuretic peptide

Values in bold indicate statistical significance

**Table 2:** Echocardiographic findings within two groups: patients diagnosed with transthyretin amyloidosis (group 1), and those without transthyretin amyloidosis (group 2)

Variable	Group 1 * (n = 12)	Group 2 * (n = 88)	p Value
Left ventricle intraventricular septum thickness (mm)	20 ± 2	17 ± 4	**0.002**
Left ventricle posterior wall thickness (mm)	17 ± 4	14 ± 3	**0.03**
Left ventricle maximum wall thickness (mm)	22 ± 3	18 ± 4	**0.002**
LV mass index (g/m^2^)	217 ± 39	162 ± 42	** < 0.001**
Left ventricle end-diastolic diameter (mm)	45 ± 8	47 ± 8	0.41
Left ventricle end-diastolic volume (ml)	102 ± 48	101 ± 48	0.79
Left atrium area (cm^2^)	32 ± 8	28 ± 11	0.71
Left atrium volume index (ml/m^2^)	61 ± 30	44 ± 21	0.76
Left ventricular ejection fraction (%)	41 ± 12	60 ± 15	** < 0.001**
Stroke volume (ml)	59 ± 21	54 ± 32	0.13
Left ventricular VTI (cm)	19 ± 6	20 ± 10	0.08
Global longitudinal strain (GLS, -%)	10 ± 5	17 ± 5	**0.008**
E/A	2.1 ± 0.7	0.9 ± 0.8	**0.005**
E/E′	19 ± 12	12 ± 7	**0.04**
Left ventricle lateral wall TDI S′ (cm/s)	4 ± 1	6 ± 3	**0.007**
Left ventricle lateral wall TDI E′ (cm/s)	6 ± 3	7 ± 5	0.12
Left ventricle lateral wall TDI A′ (cm/s)	4 ± 2	7 ± 3	**0.007**
Left ventricle intraventricular septum TDI S′ (cm/s)	4 ± 2	6 ± 3	**0.02**
Left ventricle intraventricular septum TDI E′ (cm/s)	4 ± 2	5 ± 3	0.06
Left ventricle intraventricular septum TDI A′ (cm/s)	3 ± 2	6 ± 3	**0.007**
Right ventricle thickness (mm)	8 ± 2	6 ± 2	** < 0.001**
Right ventricle outflow tract diameter (mm)	33 ± 5	33 ± 5	0.14
Right atrium area (cm^2^)	28 ± 5	20 ± 10	** < 0.001**
Right atrium volume (ml)	99 ± 21	49 ± 83	**0.04**
Right ventricle VTI (cm)	10 ± 5	16 ± 7	**0.02**
TAPSE (mm)	12 ± 5	22 ± 7	** < 0.001**
sPAP (mmHg)	36 ± 15	30 ± 12	0.06
Right ventricle TDI S′ (cm/s)	8 ± 11	11 ± 5	**0.004**
Right ventricle TDI E′ (cm/s)	8 ± 3	9 ± 3	0.66
Right ventricle TDI A′ (cm/s)	8 ± 4	13 ± 5	**0.02**
Apical sparing	11 (92%)	13 (15%)	** < 0.001**
Ground glass appearance of myocardium	12 (100%)	31 (35%)	** < 0.001**
Thickening of interatrial septum	11 (92%)	27 (30%)	** < 0.001**
Thickening of valves	12 (100%)	30 (34%)	** < 0.001**
Right ventricle thickening	12 (100%)	36 (41%)	** < 0.001**
“5–5–5” sign (s′ [systolic], e′ [early diastolic], and a′ [late (atrial) diastolic] tissue velocities are all < 5 cm/s)	6 (50%)	6 (7%)	** < 0.001**
Pericardial effusion (mm)	6 ± 3	3 ± 2	0.11

*The data is given as a number (percentage) for categorical data, and as a mean value ± one standard deviation or median (IQR) for continuous variables

*cm* centimeter, *LV* left ventricle, *mmHg* millimeters of mercury, *ml* milliliter, *mm* millimeter, *TAPSE* tricuspid annular plane systolic excursion, *TDI* tissue Doppler imaging, *s* second, *sPAP* systolic pulmonary artery pressure, *VTI* velocity time integral

Values in bold indicate statistical significance

**Table 3:** Scintigraphic assessment of the patients within two groups (group 1—patients with transthyretin amyloidosis, group 2—patients without transthyretin amyloidosis)

Variable	Group 1 * (n = 12)	Group 2 * (n = 88)	p Value
Present [99mTc]Tc-DPD tracer uptake in cardiac region	12 (100%)	1 (1%)	** < 0.001**
Perugini semi-quantitative grade 0 1 2 3	0% 0% 1 (8%) 11 (92%)	87 (99%) 0% 0% 1 (1%)	** < 0.001**
Dose (MBq)	607 ± 45	595 ± 46	0.63

*The data is given as a number (percentage) for categorical data, and as a mean value ± one standard deviation or median (IQR) for continuous variables

*MBq* Megabecquerel

Values in bold indicate statistical significance

Based on the tests performed, 18% of patients were diagnosed with CA, including five cases of light-chain amyloidosis (AL), 12 cases of ATTR, and 1 case of type A amyloidosis. Biopsies were performed in 13% of the patients. Patients with ATTR presented with higher NYHA class, and elevated levels of high sensitivity troponin (hsT) and N-terminal pro-brain natriuretic peptide (NT-proBNP) (p < 0.05). Echocardiographic evaluation revealed greater values of LV maximum wall thickness (22 ± 3 vs. 18 ± 4 mm, p = 0.002), LV mass index (217 ± 39 vs. 162 ± 42 g/m^2^, p = < 0.001), reduced LVEF (41 ± 12 vs. 60 ± 15%, < 0.001) and GLS (− 10 ± 5% vs. − 17 ± 5%, p = 0.008), as well as more advanced diastolic dysfunction (E/e′ 19 ± 12 vs. 12 ± 7, p = 0.04). Moreover, patients with ATTR exhibited a significantly greater thickness of the right ventricle (RV) wall (8 ± 2 vs. 6 ± 2), as well as increased dimensions for the right atrium area and volume. These findings were accompanied by a notable decline in RV systolic function (tricuspid annular plane systolic excursion (TAPSE), Tissue Doppler Imaging (TDI) RV S′, and RV outflow tract (RVOT) velocity time integral (VTI) (p < 0.05). Using echocardiography, it was found that patients with ATTR more frequently presented with distinct characteristics such as ‘apical sparing’, a ground glass appearance of the myocardium, thickening of interatrial septum, and thickening of the valves. In addition, the presence of the “5–5–5” sign, wherein tissue velocities {‘s’ [systolic], e′ [early diastolic], and a′ [late (atrial) diastolic]} measured in TDI were all below 5 cm/s, was also observed more frequently in patients with ATTR (p < 0.05).

ATTR was the predominant form of CA observed in this study, accounting for the majority of cases (8% ATTRwt and 4% ATTRm). In a single male patient, a pathogenic variant c.157 T > C p. (Phe53Leu) and a variant of uncertain significance (VUS) c.302C > T p. (Ala101Val) were detected. Patients with ATTR presented with a higher prevalence of polyneuropathy, gastropathy, carpal tunnel syndrome, lumbar spine stenosis, and recent weight loss over the previous 6 months (p < 0.05). Furthermore, the ATTR group exhibited a higher incidence of low voltage on ECG, as well as higher ventricular arrhythmia and greater atrioventricular block burden on Holter monitoring (p < 0.05).

**Fig. 2:**
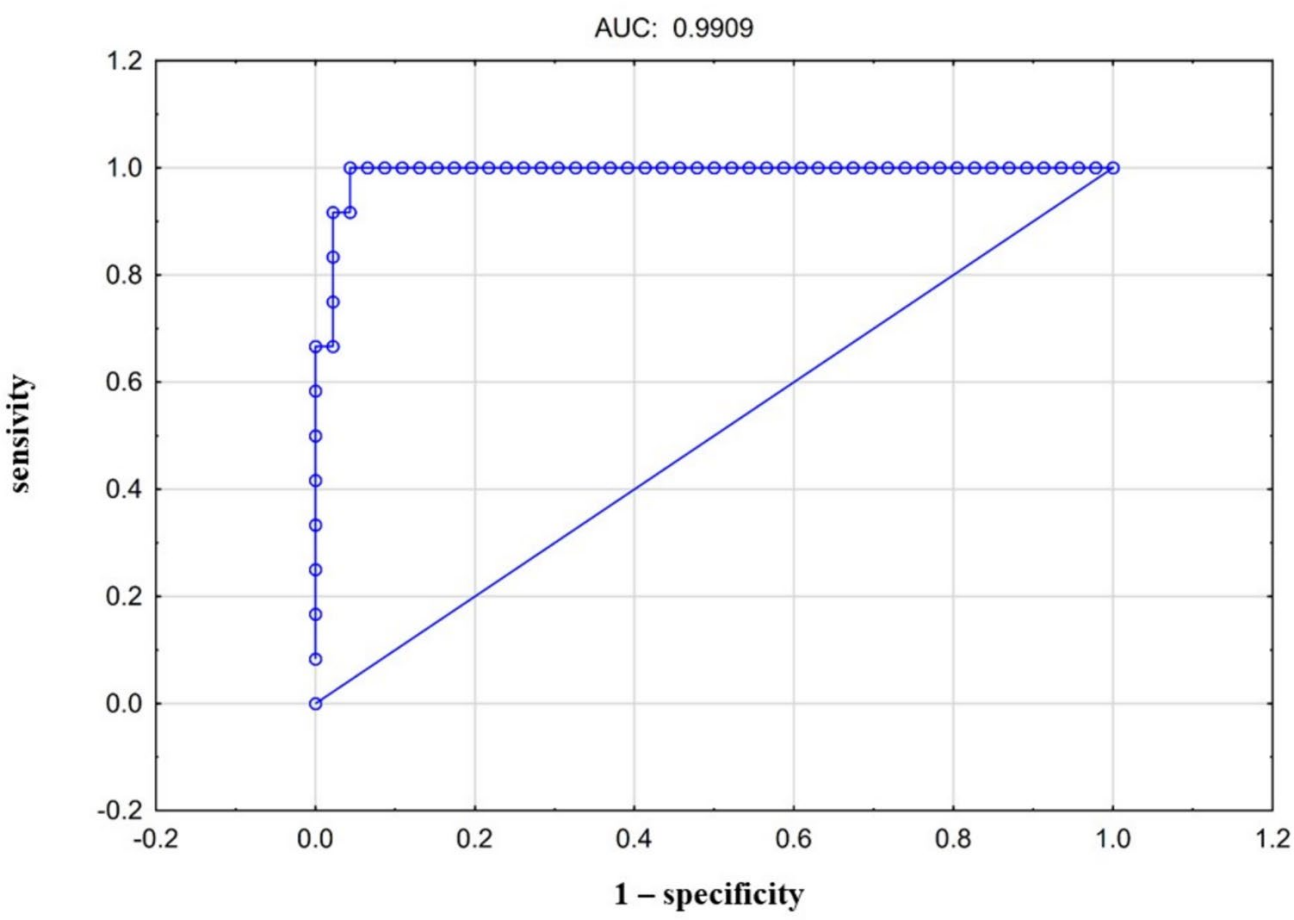
A multivariate analysis was performed to identify associations of variables with the final transthyretin amyloidosis diagnosis. The receiver operating characteristic (ROC) curve of the best model includes left ventricle mass, global longitudinal strain, and the thickness of the right ventricle

A LOGIT model was applied to calculate OR and 95% CIs for the primary endpoint, defined as ATTR diagnosis (Table 4). A multivariate analysis was performed to identify associations of variables with the final ATTR diagnosis. Overall, age, polyneuropathy, gastropathy, carpal tunnel syndrome, lumbar spine stenosis, systolic blood pressure, low voltage, ventricular arrhythmia, LV maximum wall thickness, LV mass, LV ejection fraction, GLS, E/A, E/E′, RV thickness, right atrium area, RV VTI, TAPSE, apical sparing, ground glass appearance of myocardium, thickening of interatrial septum, thickening of valves, “5–5–5” sign were associated with ATTR (p < 0.05). The ROC curve of the best model presenting the AUC value of 0.99 includes left ventricle mass, global longitudinal strain, and thickness of right ventricle (Fig. 2). The threshold value is set at 0.15 with Youden’s index (sensitivity plus specificity minus one) at 0.96, signifying an estimated sensitivity of 100% (95% CI 74 to 100%) and specificity of 96% (95% CI 85 to 99%) (Table 5*).*

**Table 4:** A generalized linear model (LOGIT) was applied to examine the associations with the primary endpoint

Variable	OR *	95% CI *	p Value *
Age	1.08	1.01–1.14	**0.009**
Polyneuropathy	21	3.32–132.99	**0.001**
Gastropathy	63.74	3.05–1329.8	**0.007**
Carpal tunnel syndrome	63.74	3.05–1329.8	**0.007**
Lumbar spine stenosis	42.5	4.22–427.34	**0.001**
Systolic blood pressure	0.96	0.93–0.99	**0.02**
NT-proBNP	1.0	0.99–1.0	0.18
Cardiac troponin T	9.21	0.02–3600.4	0.47
ECG—low voltage	19.14	4.58–80.04	** < 0.001**
Ventricular arrhythmia	37.06	4.43–310.06	** < 0.001**
LV maximum wall thickness	1.24	1.06–1.45	**0.007**
LV mass	1.02	1.01–1.03	**0.002**
LV ejection fraction	0.93	0.89–0.97	**0.002**
Global longitudinal strain	0.82	0.72–0.95	**0.007**
E/A	1.96	1.08–3.52	**0.02**
E/E′	1.1	1.03–1.18	**0.006**
Right ventricle thickness	2.62	1.5–4.57	** < 0.001**
Right atrium area	1.06	1.01–1.12	**0.03**
RV VTI	0.88	0.77–0.99	**0.01**
TAPSE	0.85	0.77–0.94	** < 0.001**
Apical sparing	50.77	6.01–428.55	** < 0.001**
Ground glass appearance of the myocardium	34.52	1.97–605.07	**0.02**
Thickening of interatrial septum	18.74	2.29–153.28	**0.006**
Thickening of valves	30.74	1.75–540.43	**0.02**
Right ventricle thickening	22.26	1.27–391.04	**0.03**
“5–5–5” sign	9	2.19–36.91	**0.002**

*Odds ratios (OR) and 95% confidence intervals (CI)

Abbreviations are listed in the Tables 1 and 2 legends

Values in bold indicate statistical significance

**Table 5:** A multivariate analysis was performed to identify associations of variables with the final transthyretin amyloidosis diagnosis. The best model includes left ventricle mass, global longitudinal strain, and the thickness of the right ventricle

Variable	Estimator	95% CI lower bound	95% CI upper bound	p Value
Intercept	− 22.6344	− 38.5115	− 6.75741	0.005204
Right ventricle wall thickness (mm)	2.4805	0.6248	4.33617	0.008796
Left ventricle mass (g)	0.0279	0.0030	0.05284	0.028067
Global longitudinal strain (-%)	− 0.4035	− 0.7781	− 0.02884	0.034787
$\text{P}=\frac{1}{1+{\text{e}}^{-({\text{a}}_{0}+{\text{a}}_{1}{\text{x}}_{1}+{\text{a}}_{2}{\text{x}}_{2}+{\text{a}}_{3}{\text{x}}_{3})}}$ $\text{P}$-prognosis ${\text{a}}_{0}=-22.6344$ ${\text{a}}_{1}=2.4805$ ${\text{a}}_{2}=0.0279$ ${\text{a}}_{3}=-0.4035$ ${\text{x}}_{1}-$ value of right ventricle wall thicknes (mm) ${\text{x}}_{2}-$ value of left ventricle mass (g) ${\text{x}}_{3}-$ value of global longitudinal strain (-%)				

*Abbreviations are listed in the Tables 1 and 2 legends

## Discussion

Amyloidosis is a severe, progressive, infiltrative disease developing secondary to the deposition of congophilic fibrils composed of aggregated misfolded proteins [20]. Recent data shows that ATTR accounts for the vast number of CA cases, although there are slight variations in disease presentation and prevalence across different geographical and ethnic populations [6, 21–23]. In this study, we identified rare types of ATTR variants. Worldwide, the most frequent variants include Val122Ile, occurring in 3.4% of African Americans [24], while in European countries, in the THAOS registry, Val30Met is the most frequent pathogenic variant, with a high concentration in Portugal; this is followed by Ile68Leu, with the highest number of cases being seen in Italy [25].

Our study employed a non-invasive approach as the primary diagnostic strategy for ATTR. In recent years, imaging techniques have made major advancements and have gained wider clinical acceptance in the diagnosis of CA [5, 26, 27]. Alongside the growing evidence supporting non-invasive diagnostic algorithms for ATTR, novel therapies have emerged, leading to improved survival rates [12–16]. Importantly, recent data has demonstrated that the prevalence of ATTR may be higher in specific patient subgroups, namely HFpEF, elderly patients, and individuals with severe AS [7, 8]. Our findings confirm that ATTR may be underdiagnosed in patients with unexpected increased LV wall thickness, emphasizing the importance of considering ATTR as a potential etiology in these cases.

In a population-based cohort study involving patients with HFpEF, aged 60 years or older, 286 individuals underwent technetium-99 m pyrophosphate ([99mTc]Tc-PYP) cardiac scintigraphy. Among this cohort, 18 patients (6.3%) were diagnosed with ATTR, and the prevalence of ATTR increased to 21% in patients aged 90 years and older (P < 0.001) [28]. Similarly, a prospective study of 120 consecutive HFpEF patients aged 60 years or older with LVH (≥ 12 mm) demonstrated uptake in 16 patients (13.3%) on [99mTc]Tc-DPD scintigraphy. Based on further clinical evaluation, the authors concluded that ATTRwt is an underdiagnosed condition that accounts for a significant number (13%) of HFpEF cases in the elderly population [7]. These findings are consistent with the prevalence of ATTR observed in this study, Moreover, autopsy studies conducted in elderly subjects have revealed the presence of amyloid deposits within the LV in 14–25% of individuals, depending on patient selection and the methodology adopted [29–32]. Conversely, the prevalence of ATTRwt in individuals aged 75 years or older, with no previous clinical suspicion of amyloidosis, was investigated over a 7-year period [33]. The prevalence of cardiac uptake was 3.88% in males and 0.77% in females, with an age-related increase reaching 13.9% in males aged 85 years or older, and 2.7% in females. In this study, the authors estimated the prevalence for the European standard population aged 75 years or older was 4.15% in males, 1.03% in females, and 2.59% in the general population.

In our study, we emphasize the diagnostic challenges of cardiac amyloidosis, highlighted by a patient case of AL amyloidosis with a positive DPD scan, definitively confirmed through histopathological examination. This case underscores the essential nature of a thorough and multi-disciplinary diagnostic approach in accurately diagnosing and treating this multifaceted condition.

Importantly, ATTR has been reported in patients with AS [8, 11]. A study examining 151 elderly patients with severe symptomatic AS, who underwent transcatheter aortic valve replacement (TAVR), investigated the presence of ATTR using [99mTc]Tc-PYP cardiac scintigraphy. The results revealed that 16% of the patients screened positive for ATTR [8]. Clinically, ATTR was associated with a severe AS phenotype characterized by low-flow, low-gradient with mildly reduced ejection fraction, more pronounced LV thickening, advanced diastolic dysfunction, and more impaired global longitudinal strain (P < 0.05). The average of lateral and septal mitral annular TDI S′ was found to be the best predictor of ATTR. Notably, a TDI S′ value < 6 cm/s conferred 100% sensitivity for predicting a positive [99mTc]Tc-PYP amyloid scan. In our study, patients with ATTR exhibited significantly lower TDI S′ and A′ velocities in both the LV lateral wall and intraventricular septum (p < 0.05). In this study, patients presenting with severe AS were systematically excluded from the analysis to delineate more clearly the phenotypic characteristics and prevalence of ATTR cardiomyopathy within a cohort presenting with unexplained left ventricular hypertrophy. This decision was predicated on the objective to reduce confounding factors attributable to the hemodynamic and structural impacts of AS on the heart, which can mimic or obscure the manifestations of ATTR cardiomyopathy. The exclusion of this subgroup allows for a more precise characterization of ATTR-related cardiac changes, offering clarity in the context of this specific cardiac amyloidosis. However, this methodological choice warrants a discussion regarding its potential impact on the study’s findings and the generalizability of the results. The exclusion of patients with severe AS might lead to an underestimation of the true prevalence of ATTR cardiomyopathy. This is particularly relevant considering that ATTR and AS can coexist, especially in the elderly population, where both conditions are more prevalent. The concurrent presence of AS might not only contribute to the LV hypertrophy observed in ATTR cardiomyopathy but could also serve as a confounding factor leading to underdiagnosis or misdiagnosis of the condition.

Despite the common occurrence of increased LV wall thickness in routine clinical practice, limited data exists regarding the frequency with which ATTR contributes to these lesions. Given the ongoing development of novel therapeutic agents targeting ATTR, it is important to establish a comprehensive understanding of the prevalence of this disease in clinical settings. In a prospective multicenter study, the TTR gene was sequenced in a cohort of patients with LVH, revealing 5% exhibited ATTRm-related CA [9]. Subsequently, an adjusted multivariate model demonstrated that African origin, neuropathy, carpal tunnel syndrome, ECG low voltage, and late gadolinium enhancement (LGE) at cardiac-magnetic resonance imaging were identified as independent factors associated with ATTRm. Similarly, in our study, neuropathy, carpal tunnel syndrome, and ECG low voltage were also significantly associated with the diagnosis of ATTR. Interestingly, hypertension was found to be present in 50% of the patients, suggesting that the frequency of ATTRm in that particular group could be underestimated in comparison to populations where individuals with any preexisting cause of LV hypertrophy were excluded.

*In choosing a left ventricular thickness threshold of 15 mm for inclusion, we aimed to focus on patients potentially exhibiting ATTR cardiomyopathy who present as hypertrophic cardiomyopathy phencopy, distinguishing our cohort from the more general group of patients with ‘any’ hypertrophy (that can be also defined as LV wall thickness* > 12 mm). This decision allows for a targeted analysis of ATTR-specific manifestations, acknowledging that a 12 mm threshold often serves as a general red flag for cardiac amyloidosis but may include a wider range of conditions and disease severities.

Our analysis data shows that a significant proportion of patients with unexplained LV thickening are likely affected by cardiac amyloidosis (CA). This finding supports previous reports in the literature suggesting the probable occurrence of this underdiagnosed condition [9]. Considering the critical role of appropriate pharmacological treatment in managing patients with AL and ATTR, it becomes imperative to grasp the implications of CA on the therapeutic pathway. The challenge also lies in accurately interpreting complementary molecular imaging-based diagnostic approaches. Our analysis identifies several factors associated with ATTR, including polyneuropathy, gastropathy, carpal tunnel syndrome, lumbar spine stenosis, low voltage, ventricular arrhythmia, LV maximum wall thickness, LV mass, LV ejection fraction, GLS, E/A, E/E′, RV thickness, right atrium area, RV VTI, TAPSE, apical sparing, ground glass appearance of myocardium, thickening of interatrial septum, thickening of valves, and the “5–5–5” sign. Moreover, ATTR appears particularly likely when increased LV mass coincides with decreased GLS and thickening of RV. Therefore, it follows that patients exhibiting these clinical characteristics should qualify for rigorous medical scrutiny during the interpretation of imaging results.

## Limitations

This study was carried out in a cardiology reference center, specialized in heart failure and cardiomyopathies. Thus, this might have been a source of selection bias stemming from the referrals of preselected patients. Since this study was a single-center project, the results need to be validated in a multicenter setting. Due to the local demographics, all the participants in this study were Caucasian. Thus, extrapolating results to more distant geographical areas may be suboptimal. Moreover, the protocol was based on primarily non-invasive approaches and scintigraphic evaluation. Performing soft tissue and/or endomyocardial biopsy was performed solely on those patients with unequivocal results from the non-invasive algorithm [4]. Though this methodology has clear limitations, it has still been adopted by leading experts in the field [4–6].

## Conclusions

In recent years, scintigraphy has increasingly gained recognition as a valuable complementary imaging technique in the diagnostic algorithm for amyloidosis. Crucially, our study highlights that a significant number of patients with unexplained increased LV wall thickness, evaluated in a cardiology referral center, may suffer from underlying CA. The identified TTR gene variants associated with ATTR included the following variants: Phe53Leu, and Ala101Val. Based on our findings, it is vital to consider the possibility of ATTR in patients with increased LV mass, accompanied by decreased GLS and thickening of the RV.

## References

[1] Arbelo E, Protonotarios A, Gimeno JR et al (2023) ESC Guidelines for the management of cardiomyopathies. Eur Heart J 44(37):3503–3626. 10.1093/eurheartj/ehad194 37622657 10.1093/eurheartj/ehad194

[2] Ommen SR, Mital S, Burke MA, Day SM, Deswal A, Elliott P et al (2020) 2020 AHA/ACC guideline for the diagnosis and treatment of patients with hypertrophic cardiomyopathy: a report of the American College of Cardiology/American Heart Association Joint Committee on Clinical Practice Guidelines. Circulation 142:e558–e631. 10.1161/CIR.0000000000000937 33215931 10.1161/CIR.0000000000000937

[3] Westermark P, Benson MD, Buxbaum JN, Cohen AS, Frangione B, Ikeda S et al (2005) Nomenclature committee of the international society of amyloidosis. Amyloid: toward terminology clarification. Report from the nomenclature committee of the international society of amyloidosis. Amyloid 12:1–4. 10.1080/13506120500032196 16076605 10.1080/13506120500032196

[4] Gillmore JD, Maurer MS, Falk RH, Merlini G, Damy T, Dispenzieri A et al (2016) Nonbiopsy diagnosis of cardiac transthyretin amyloidosis. Circulation 133:2404–2412. 10.1161/CIRCULATIONAHA.116.021612 27143678 10.1161/CIRCULATIONAHA.116.021612

[5] Dorbala S, Ando Y, Bokhari S, Dispenzieri A, Falk RH, Ferrari VA et al (2019) ASNC/AHA/ASE/EANM/HFSA/ISA/SCMR/SNMMI expert consensus recommendations for multimodality imaging in cardiac amyloidosis: part 1 of 2-evidence base and standardized methods of imaging. J Nucl Cardiol 26:2065–2123. 10.1007/s12350-019-01760-6 31468376 10.1007/s12350-019-01760-6

[6] Garcia-Pavia P, Rapezzi C, Adler Y, Arad M, Basso C, Brucato A et al (2021) Diagnosis and treatment of cardiac amyloidosis: a position statement of the ESC working group on myocardial and pericardial diseases. Eur Heart J 42:1554–1568. 10.1093/eurheartj/ehab072 33825853 10.1093/eurheartj/ehab072 PMC8060056

[7] González-López E, Gallego-Delgado M, Guzzo-Merello G, de Haro-Del Moral FJ, Cobo-Marcos M, Robles C et al (2015) Wild-type transthyretin amyloidosis as a cause of heart failure with preserved ejection fraction. Eur Heart J 36:2585–2594. 10.1093/eurheartj/ehv338 26224076 10.1093/eurheartj/ehv338

[8] Castaño A, Narotsky DL, Hamid N, Khalique OK, Morgenstern R, DeLuca A et al (2017) Unveiling transthyretin cardiac amyloidosis and its predictors among elderly patients with severe aortic stenosis undergoing transcatheter aortic valve replacement. Eur Heart J 38:2879–2887. 10.1093/eurheartj/ehx350 29019612 10.1093/eurheartj/ehx350 PMC5837725

[9] Damy T, Costes B, Hagège AA, Donal E, Eicher JC, Slama M et al (2016) Prevalence and clinical phenotype of hereditary transthyretin amyloid cardiomyopathy in patients with increased left ventricular wall thickness. Eur Heart J 37:1826–1834. 10.1093/eurheartj/ehv583 26537620 10.1093/eurheartj/ehv583

[10] Petkow-Dimitrow P, Rajtar-Salwa R, Holcman K, Kostkiewicz M, Rubiś P (2020) From hypertrophic cardiomyopathy to transthyretin amyloidosis: an unusual case and challenging diagnosis. Pol Arch Intern Med 130:153–154. 10.20452/pamw.15140 31933485 10.20452/pamw.15140

[11] Merli E, Giudice ED, Antonopoulos A, Amadei G, Varani E (2017) Transthyretin cardiac amyloid and aortic stenosis in the elderly, the role of nuclear imaging. Int J Cardiovasc Imaging 33:947–949. 10.1007/s10554-016-1060-4 28155044 10.1007/s10554-016-1060-4

[12] Coelho T, Maia LF, Martins da Silva A, Waddington Cruz M, Planté-Bordeneuve V, Lozeron P et al (2012) Tafamidis for transthyretin familial amyloid polyneuropathy: a randomized, controlled trial. Neurology 79:785–792. 10.1212/WNL.0b013e3182661eb1 22843282 10.1212/WNL.0b013e3182661eb1 PMC4098875

[13] Adams D, Gonzalez-Duarte A, O’Riordan WD, Yang CC, Ueda M, Kristen AV et al (2018) Patisiran, an RNAi therapeutic, for hereditary transthyretin amyloidosis. N Engl J Med 379:11–21. 10.1056/NEJMoa1716153 29972753 10.1056/NEJMoa1716153

[14] Minamisawa M, Claggett B, Adams D, Kristen AV, Merlini G, Slama MS et al (2019) Association of Patisiran, an RNA interference therapeutic, with regional left ventricular myocardial strain in hereditary transthyretin amyloidosis: the APOLLO study. JAMA Cardiol 4:466–472. 10.1001/jamacardio.2019.0849 30878017 10.1001/jamacardio.2019.0849 PMC6537803

[15] Solomon SD, Adams D, Kristen A, Grogan M, González-Duarte A, Maurer MS et al (2019) Effects of Patisiran, an RNA interference therapeutic, on cardiac parameters in patients with hereditary transthyretin-mediated amyloidosis. Circulation 139:431–443. 10.1161/CIRCULATIONAHA.118.035831 30586695 10.1161/CIRCULATIONAHA.118.035831 PMC12611557

[16] Benson MD, Waddington-Cruz M, Berk JL, Polydefkis M, Dyck PJ, Wang AK et al (2018) Inotersen treatment for patients with hereditary transthyretin amyloidosis. N Engl J Med 379:22–31. 10.1056/NEJMoa1716793 29972757 10.1056/NEJMoa1716793 PMC12611561

[17] Lang RM, Badano LP, Mor-Avi V, Afilalo J, Armstrong A, Ernande L et al (2015) Recommendations for cardiac chamber quantification by echocardiography in adults: an update from the American Society of Echocardiography and the European Association of Cardiovascular Imaging. J Am Soc Echocardiogr 28:1-39.e14. 10.1016/j.echo.2014.10.003 25559473 10.1016/j.echo.2014.10.003

[18] Phelan D, Collier P, Thavendiranathan P, Popović ZB, Hanna M, Plana JC et al (2012) Relative apical sparing of longitudinal strain using two-dimensional speckle-tracking echocardiography is both sensitive and specific for the diagnosis of cardiac amyloidosis. Heart 98:1442–1448. 10.1136/heartjnl-2012-302353 22865865 10.1136/heartjnl-2012-302353

[19] Perugini E, Guidalotti PL, Salvi F, Cooke RM, Pettinato C, Riva L et al (2005) Noninvasive etiologic diagnosis of cardiac amyloidosis using 99mTc-3,3-diphosphono-1,2-propanodicarboxylic acid scintigraphy. J Am Coll Cardiol 46:1076–1084. 10.1016/j.jacc.2005.05.073 16168294 10.1016/j.jacc.2005.05.073

[20] Merlini G, Bellotti V (2003) Molecular mechanisms of amyloidosis. N Engl J Med 349:583–596. 10.1056/NEJMra023144 12904524 10.1056/NEJMra023144

[21] Damy T, Deux JF, Moutereau S, Guendouz S, Mohty D, Rappeneau S et al (2013) Role of natriuretic peptide to predict cardiac abnormalities in patients with hereditary transthyretin amyloidosis. Amyloid 20:212–220. 10.3109/13506129.2013.825240 23964755 10.3109/13506129.2013.825240

[22] Mróz K, Rubiś P, Podolec P, Kostkiewicz M, Holcman K (2023) Multimodality family screening of patients with cardiac transthyretin amyloidosis: a case of an asymptomatic patient. Eur Heart J Case Rep. 10.1093/ehjcr/ytad200 37197210 10.1093/ehjcr/ytad200 PMC10184687

[23] Gawor M, Holcman K, Franaszczyk M, Lipowska M, Michałek P, Teresińska A et al (2022) Spectrum of transthyretin gene mutations and clinical characteristics of polish patients with cardiac transthyretin amyloidosis. Cardiol J 29:985–993. 10.5603/CJ.a2020.0104 32789836 10.5603/CJ.a2020.0104 PMC9788745

[24] Jacobson DR, Pastore RD, Yaghoubian R, Kane I, Gallo G, Buck FS et al (1997) Variant-sequence transthyretin (isoleucine 122) in late-onset cardiac amyloidosis in Black Americans. N Engl J Med 336:466–473. 10.1056/NEJM199702133360703 9017939 10.1056/NEJM199702133360703

[25] Damy T, Kristen AV, Suhr OB, Maurer MS, Planté-Bordeneuve V, Yu CR et al (2019) Transthyretin cardiac amyloidosis in continental Western Europe: an insight through the transthyretin amyloidosis outcomes survey (THAOS). Eur Heart J 43:391–400. 10.1093/eurheartj/ehz173 10.1093/eurheartj/ehz173 PMC8825236 30938420

[26] Holcman K, Kostkiewicz M, Podolec P, Rubiś P (2019) Cardiac amyloidosis—state-of-the-art diagnosis and emerging therapies. Folia Cardiol 14:616–624. 10.5603/FC.2019.0115 10.5603/FC.2019.0115

[27] Holcman K, Dziuk M, Grzybowski J, Teresinska A, Malkowski B, Jedrzejuk D et al (2022) The scintigraphic diagnosis of cardiac amyloidosis. An expert opinion endorsed by the Section of nuclear medicine of the polish cardiac society and the polish nuclear medicine society. Nucl Med Rev Cent East Eur 25:142–147. 10.5603/NMR.a2022.0033 35929128 10.5603/NMR.a2022.0033

[28] AbouEzzeddine OF, Davies DR, Scott CG, Fayyaz AU, Askew JW, McKie PM et al (2021) Prevalence of transthyretin amyloid cardiomyopathy in heart failure with preserved ejection fraction. JAMA Cardiol 6:1267–1274. 10.1001/jamacardio.2021.3070 34431962 10.1001/jamacardio.2021.3070 PMC8387947

[29] Tanskanen M, Peuralinna T, Polvikoski T, Notkola IL, Sulkava R, Hardy J et al (2008) Senile systemic amyloidosis affects 25% of the very aged and associates with genetic variation in alpha2-macroglobulin and tau: a population-based autopsy study. Ann Med 40:232–239. 10.1080/07853890701842988 18382889 10.1080/07853890701842988

[30] Mohammed SF, Mirzoyev SA, Edwards WD, Dogan A, Grogan DR, Dunlay SM et al (2014) Left ventricular amyloid deposition in patients with heart failure and preserved ejection fraction. JACC Heart Fail 2:113–122. 10.1016/j.jchf.2013.11.004 24720917 10.1016/j.jchf.2013.11.004 PMC3984539

[31] Hodkinson HM, Pomerance A (1977) The clinical significance of senile cardiac amyloidosis: a prospective clinico-pathological study. Q J Med 46:381–387. 10.1093/oxfordjournals.qjmed.a067513 918253 10.1093/oxfordjournals.qjmed.a067513

[32] Lie JT, Hammond PI (1988) Pathology of the senescent heart: anatomic observations on 237 autopsy studies of patients 90 to 105 years old. Mayo Clin Proc 63:552–564. 10.1016/S0025-6196(12)64885-X 3374172 10.1016/S0025-6196(12)64885-X

[33] Mohamed-Salem L, Santos-Mateo JJ, Sanchez-Serna J, Hernández-Vicente Á, Reyes-Marle R, Castellón Sánchez MI et al (2018) Prevalence of wild type ATTR assessed as myocardial uptake in bone scan in the elderly population. Int J Cardiol 270:192–196. 10.1016/j.ijcard.2018.06.006 29903517 10.1016/j.ijcard.2018.06.006

